# Effect of Four Grape Varieties on the Physicochemical and Sensory Properties of Unripe Grape Verjuice

**DOI:** 10.1155/2020/6457982

**Published:** 2020-07-12

**Authors:** Najiba Salah Eddine, Sami Tlais, Ali Alkhatib, Rasha Hamdan

**Affiliations:** ^1^Lebanese International University, Nutrition and Food Sciences Department, P.O. Box 5, Jeb-jenine Bekaa, Lebanon; ^2^Lebanese University, Faculty of Science, Lebanon

## Abstract

Verjuice is a sour-tasting juice obtained from the mechanical pressing of unripe grapes. The significance of verjuice as food product includes but not limited to its richness in antioxidant compounds, its usage as an alternative to lemon and vinegar, and also its production which can reduce the losses of lower quality grapes and waste from grape thinning. In this study, a survey for the common Lebanese traditional preparation methods for verjuice was done and physicochemical properties of four Lebanese verjuice varieties Tfayfihi, Baytamoni, Black, and Obeideh along with their sensory evaluation by consumers were studied. Results showed that “Black” grape verjuice has the highest density (1.01 ± 0.003 g/L), titratable acidity (4.51 g/L ± 0.03), total soluble solids (5.38°Brix ± 0.3), and polyphenol content (676.1 mg/L ± 6.8); verjuice processed from the Baytamoni grape variety has the highest browning index (0.432 ± 0.002) and color intensity (1.18 ± 0.007); “Obeideh” grape verjuice has the highest pH (2.55 ± 0.006); and “Tfayfihi” grape verjuice has the highest radical scavenging potential (91.76% ± 0.43) and moisture content (95.85% ± 0.19). Both “Tfayfihi” and “Black” grape verjuice has the highest total suspended solids (40 g/L ± 1.3 and 40 g/L ± 2.9, respectively) among all studied verjuice. There is no difference in taste between the four verjuice varieties which we studied, but there is a color preference for the “Tfayfihi” verjuice. The use of different varieties of grapes in the processing of verjuice affects the physicochemical and sensory properties and results in selection of grape varieties being favorable in the processing of verjuice with respect to factors such as polyphenol content and color of the final product.

## 1. Introduction

Verjuice is an acidic tart flavor juice obtained from pressing and processing of unripe grape clusters. Verjuice is mainly consumed in the Mediterranean region, such as Italy, Turkey, and Iran, and it is gaining more popularity in western countries [1]. It is called “Verjuice” in French, “Agraz” in Spanish, “Agresto” in Italian, “Koruk” in Turkish, and “Abbe-ghureh” in Persian [2]. In Lebanon, verjuice is called “Asir el hosrom”, and it is usually prepared during July, at the beginning of the summer season. Unripe grapes are harvested approximately 45 days after the flowering of the grapevine [3, 4], at the end of the lag grapes' growth phase, just before the onset of veraison phase to avoid the increase of sugar concentration and the decrease in organic acid content including polyphenols [4].

There is a growing interest in verjuice consumption worldwide, not only due its potential use as an alternative to lemon juice and vinegar in adding the sour acidic flavor [1, 3, 5] but also due to its high content of polyphenolic compounds known for their antioxidative role inside the body and its low sugar content [4, 6, 7]. In ancient times, old Greeks used verjuice as a medicine to treat ulcers. It was also mentioned in early modern times in the book named “Tuhfat al Muminin” as an agent that aids the digestion of high-fat meals [8]. Verjuice was used in Iranian traditional medicine as a lipid-lowering juice that can also control hypertension and protect against atherosclerosis [4]. It is assumed to have cardioprotective properties due to its richness in bioactive compounds and its extensive phytochemical profile. It is also proposed to elicit beneficial changes to serum lipid profile, blood pressure, inflammatory markers, glycemic control, and fatty streak formation [1], and it reduces the total cholesterol with no significant reduction in LDL (Mousa-Al-Reza, 2011, [9]). In addition, verjuice has a potential role as a food preservative due to its high acidity, where it has been shown to have the ability to inhibit the growth of *Escherichia coli*, *Listeria monocytogenes*, and *Salmonella typhimurium* [4, 10, 11]. From an economical point of view, verjuice production is an efficient way to convert a vineyard waste during a thinning period into a high value product [12].

Although verjuice has promising potential in food industry, limited studies on the basic parameters that affect the standardized method of production are being done. In this study, four grapevine *Vitis vinifera* L. varieties from Bekaa area of Lebanon were processed into verjuice and the physicochemical and sensory characteristics were investigated to determine to the effect of grape variety on the properties of verjuice.

## 2. Materials and Methods

### 2.1. Production of Unripe Grape Juice

**Figure 1:**
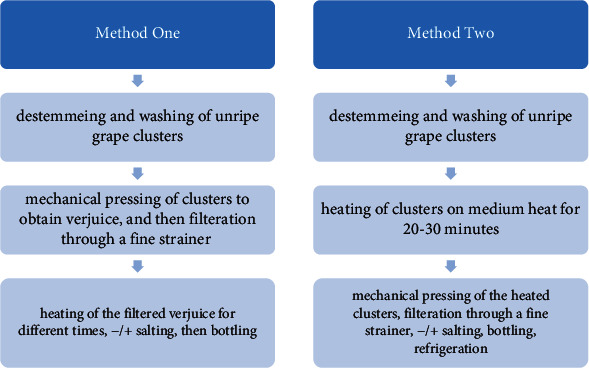
Summary for traditional Lebanese verjuice processing methods.

Four of the most common planted grapevine *Vitis vinifera* L. varieties in Lebanon were selected [13]. These varieties are Tfayfihi, Black (red grape varieties), Baytamoni, and Obeideh (white grape varieties). The unripe grapes of the four varieties were harvested in early July, 2017. Fourteen kilograms of each variety was collected from the same vineyard located in Almanarah town, West Bekaa region of Lebanon (latitude 33°56′53.48^″^ and longitude 35°53′12.78^″^). Processing method one was applied in preparation of the four verjuice samples using four varieties of unripe grapes (Figure 1). Unripe grapes were harvested before the onset of veraison; clusters were destemmed, washed, and mechanically grinded. The grounded clusters were then mechanically pressed to obtain the juice which was sieved and boiled for 30 minutes before bottling. The solution volume decreased by around 15% upon boiling and sieving. Fresh samples from the four varieties of verjuice were frozen at -20°C without boiling for later analysis.

### 2.2. Physiochemical Analysis

The physicochemical properties were done as follows: the titratable acidity of verjuice was calculated as the percentage of tartaric acid since it is the most abundant organic acid in verjuice [14, 15]. A pH/temperature microprocessor-based bench meter, equipped with digital probe 230 VAC, was used to measure the pH. A handheld refractometer (Model REF 107, 0-90% Brix) was used to measure the soluble solid content of verjuice [15]. Color intensity of all verjuice samples was measured spectrophotometrically (spectrophotometer, Thermo, model Genesys 10-S) based on the color intensity measurement of wine (*I* = (*A* at 420 nm) + (*A* at 520 nm), where *I* is the intensity and *A* is the absorbance) [16]. The Folin-Ciocalteu method for wines was adopted to find the total quantity of polyphenols in verjuice [17]. The antioxidant activity of the polyphenols found in verjuice was tested by the ability of these polyphenols to scavenge hydrogen peroxide [18, 19]. Suspended solids were determined using Method 160.2 in Methods for Chemical Analysis of Water and Wastes (USEPA, 1983). Moisture percentage was measured using a moisture analyzer (Redwag MA 210.R, 563610, Tmax 160°C). A UV-visible spectrophotometer (spectrophotometer, Thermo, model Genesys 10-S) was used for measuring the browning index [20].

### 2.3. Sensory Evaluation

Two questionnaires regarding processing and consumption of verjuice were conducted in this study. The first questionnaire assessed processing methods of verjuice by 12 verjuice processers from different villages in the Bekaa Valley of Lebanon (Appendix 1), while the second questionnaire included questions about the preference consumption over lemon juice and food uses of verjuice (Appendix 2) which was answered by 42 female panelists aged 18+.

A questionnaire was used to assess which is the preferred color of verjuice among the four processed verjuice (Appendix 3). Forty-two trained panelists participated in this questionnaire (38 females and 4 males), whereas a triangle test was performed among thirty-six trained panelists (30 females, 6 males, aged 18+).

### 2.4. Statistical Analysis

All analyses were performed with replicates. The statistical analyses were performed using the SPSS (Statistical Package for the Social Sciences, version 22.0) program.The analysis of variance which consisted of univariate analysis was performed followed by Tukey's Honest Significant Difference test to compare means between processed verjuice samples in physicochemical tests. Bivariate correlations obtained, such as Pearson's correlation coefficients (two tailed), cluster analysis, and principal component analysis, were performed for processed samples' results using SPSS.

A two-tailed *t*-test was used to compare the means of fresh and processed verjuice samples in all physicochemical tests except for the browning index because the browning reaction type occurring in fresh verjuice is different from the browning reaction type occurring in processed verjuice.

## 3. Results and Discussion

### 3.1. Verjuice Processing and Consumption Questionnaires

Processing evaluation questionnaire showed differences in mechanical pressing duration and heating duration and whether mixed or single variety of unripe grapes was used. The processing evaluation questionnaire resulted in two processing methods described in Figure 1, wherein 83% of the processors apply method one, whereas 17% apply method two.

The heating duration, selection of grape variety, and salt or oil addition are done depending on the processors' sensory preferences in terms of color, viscosity, and flavor. Different heating durations are applied by processors using processing method one, where 10% of these processors heated the verjuice for 15 minutes, 30% heated it for 30 minutes, 25% applied heating for a duration between 30 minutes to 2 hours, and 40% heated the verjuice for more than 2 hours. Only 25% of processors use single grape variety in their verjuice preparation, and the remaining processors (75%) use mixed grape varieties. Only 42% of processors add table salt to their verjuice before bottling. Compared to the traditional Turkish method of verjuice preparation, none of the Lebanese processors added olive oil to the verjuice [3].

The results of the consumption evaluation questionnaire (Appendix 2) showed that 93% of the panelists know the verjuice, while 7% panelists do not. With regard to the use of verjuice, 97% of the panelists who are already familiar to verjuice use it in their food. With respect to the priority of verjuice when both verjuice and lemon juice are available at the same time where 23% of the participants prefer verjuice over lemon juice when available, 34% of consumers prefer lemon juice over verjuice, and 43% prefer to add verjuice only to specific dishes. Out of the 43% panelists, 40% use verjuice in salad and 55% use it in sour Kibbeh (meat dish) while the remaining 5% use verjuice in other unspecified dishes. The differences in aroma, flavor compounds, and color are likely to be the reasons behind the preference of verjuice addition to some dishes. On the contrary, pH does not seem to be an important factor since both lemon and verjuice have comparable pH of 2.2 and 2.5, respectively [3, 21].

### 3.2. Physicochemical Analysis of Verjuice

The physicochemical properties of the processed and fresh sample of the four verjuice varieties are summarized in Table 1. The pH values for all fresh and processed verjuice samples were comparable and ranged from 2.52 to 2.59. Therefore, the pH of verjuice samples was not affected by heat processing in the production due to short heating time. Conversely, the average titratable acidity, which is calculated as tartaric acid percentage, increased with the processing from 3.75% in fresh samples to 4.25% in the processed verjuice, where “Black” grape sample showed the highest increase (23%) in titratable acidity upon processing. Among the fresh samples, “Obeideh” has the highest titratable acidity value (4.09%) while “Baytamoni” had the lowest (3.45%). The titratable acidity values of verjuice are close to the previous studied verjuice samples [2]. Grape variety, degree of ripeness, and climate are among the factors that contribute to the organic acid quantity in grape juice, which affects microbiological stability and taste [22] and therefore acidity and pH.

The average density of the fresh and processed verjuice was 1.01 g/cm^3^ and 1.00 g/cm^3^, respectively (Table 1). According to Nikfardjam [8], the average density of seven verjuice samples studied was 1.03 g/cm^3^.

In general, at the unripe stage of grape development, the main soluble solids are phenolic compounds, fructose, glucose, citric acid, malic, and tartaric (Sabir, 2010). Among our samples, the total soluble solids (TSS) ranged from 3.72°Brix for the Obeideh variety and 4.91°Brix for the Black variety. Similar results were reported previously; Yediveren and American Rootstock varieties of processed verjuice had total soluble solid content of 3.21°Brix and 4.28°Brix, respectively [2]. Moreover, Hayoglu et al. [3] reported a significant difference in the TSS content between the two varieties Kabarcik (7.47°Brix) and Yediveren (4.50°Brix). These differences are attributed to many factors including growing conditions, genotype of the grape vine, time of harvest, the sensitivity of each variety to the growing conditions, and delays in crop sampling. The processing of fresh verjuice samples did not result in a significant increase in the TSS in the processed samples except for Obeideh variety, and the difference in TSS was only significant between processed varieties of Black and Obeideh (Table 1). The small change in TSS content after processing can be aligned with the moisture decrease on one hand and the polyphenol content increase on the other hand.

Both total suspended solids and moisture percentage decreased significantly between the fresh samples and the processed samples of all verjuice varieties mainly due to the filtration (sieving) step and evaporation, respectively, involved in processing (Table 1).

The difference in color intensity between fresh and processed verjuice and among the processed verjuice themselves was significant; Baytamoni variety has the highest color intensity among the processed samples (*I* = 1.18) (Table 1). Visually, the fresh samples were turbid and had a dark greenish color, but after 15 minutes of boiling, the solution became clearer and changed to a yellowish color. This could be due to the aggregation by heating of some components, such as polysaccharides and protein, and result in a decreased blurriness of verjuice samples (Hayoglu, 2009). With respect to the browning index, all fresh varieties were significantly different from each other, while only the processed verjuice of Obeideh variety was significant from the other processed varieties with the lowest browning index of 0.228 nm. However, all varieties showed an increase in the browning index and color intensity after heat processing. The nonenzymatic browning reaction, known as Maillard reaction, can contribute to a part of the color change in the samples [23]. The extent to which Maillard reaction occurs in juice depends on the sugar and amino acid content of juice and heating time. Obeideh processed verjuice had the lowest total soluble solids (which include, but not limited to, sugars and amino acids) and also showed the lowest color intensity among the other processed varieties (4.45°Brix and 719). This might allow us to conclude that lower color intensity is related to a lower amount of total soluble solids undergoing Maillard reaction. Also, caramelization can be another factor for change in color where simple sugars are degraded upon heating and result in a brownish shade (Quintas, 2005). A positive linear correlation between gallic, vanillic, ferulic, and protocatechuic acid content and color intensity in posip Rukatac wines was shown by Irena (2002). Enzymatic browning reaction catalyzed by polyphenol oxidase enzyme in the presence of oxygen is another process by which color change is taking place, at least in the early stages of the process before the complete inactivation of enzymes [24].

Many polyphenols of phytochemical properties are present in grapes and their products such as gallic acid, caffeic acid, caftaric acid, catechin, epicatechin, anthocyanins, quercetin, and many others [1], of which many acts as antioxidants [2]. The polyphenols content significantly increased by 56%, 50%, and 37% in processed verjuice samples of Tfayfihi, Baytamoni, and Black varieties, respectively, compared to fresh samples of the same varieties. Obeideh variety, however, showed a nonsignificant 4% decrease in polyphenols after processing (Table 1). The increase in polyphenols content can be attributed to several factors. The evaporation process of water during the preparation of verjuice [25] is one of the most important reasons for the increase concentration of polyphenols. Another factor is the presence of suspended solids, which are traces of grape pomace [26], that released polyphenols upon heating, where Chamorro et al. explained the increase in antioxidants in tomatoes upon heating to the release of phytochemicals from the cells [27]. Another reason for the increase in polyphenols upon heating of grape juice was proposed by Piva et al. [28] through considering the behavior of two phenolic compounds: catechin and proanthocyanidins. Catechins are degraded and oxidized by heating and concentrating and therefore decrease in processed grape juice. In contrast, proanthocyanidins, condensed tannins, are low in fresh grape juices but increase in heated grape juices due to oxidation which leads to the polymerization of single polyphenols. The same process occurs during the aging of wine (Piva, 2008). The polyphenol content in processed verjuice of Baytamoni and Black varieties (667.20 mg/L and 676.10 mg/L, respectively) was not significantly different, although Baytamoni is a white variety while Black is a colored variety (Table 1). While it is well known that colored grape varieties are higher in polyphenols than in white grape varieties [29], according to Chamorro (2007), it is not easy to expect the behavior of polyphenols in the presence of heat since some types may increase and others may decrease.

In this study, the antioxidant capacity *in vitro* was evaluated by the hydrogen peroxide scavenging (Hs) method. The average Hs of Tfayfihi and Baytamoni varieties increased significantly from 68 to 91% upon processing, while Black and Obeideh varieties showed no significant change in Hs upon processing (Table 1). Previous studies have suggested that processing can promote the oxidation of polyphenols to an intermediate oxidation state, which can exhibit a higher radical scavenging efficiency than the nonoxidized polyphenols (Nicoli, 1999).

Correlation analysis indicated that only the % of moisture and titratable acidity are significantly correlated (*p* = 0.05). This correlation is negative (*r* = −0.962), meaning that the titratable acidity increased when % of moisture decreased in processed verjuice varieties. According to the results of the principal component analysis, three components were extracted: browning index, total soluble solids, and pH as the main factors that account to most of the variation between the verjuice samples.

**Figure 2:**
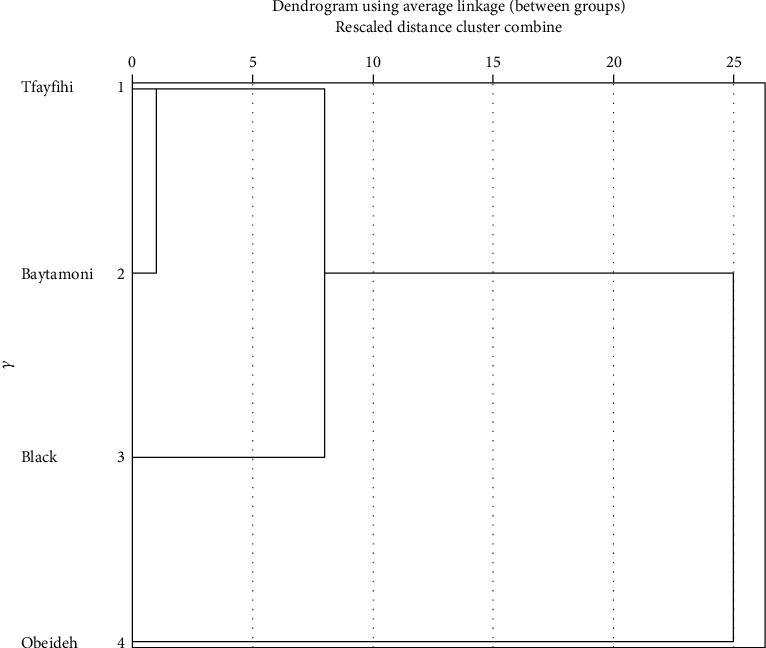
Cluster analysis dendrogram of the four verjuice varieties based on the studied physicochemical characteristics.

Cluster analysis was conducted to find which processed verjuice varieties, Tfayfihi, Baytamoni, Black, or Obeideh, are highly similar to each other in terms of physicochemical properties. The results showed that the “Tfayfihi” processed verjuice and “Baytamoni” processed verjuice have the most similar characteristics while “Obeideh” is the most distant variety (Figure 2).

### 3.3. Sensory Evaluation and Color Preference Questionnaire

The majority of taste panelists were unable to detect the different verjuice sample among the samples presented in the triangle test (significance level set for this triangle test is *α* = 0.05). This could be explained by two reasons; the first reason is that the taste panelists are not professional trained panelists experienced in acidic flavor differentiation, and the second reason is the high acidity of the processed verjuice samples with pH values being nonsignificantly different with each other. Therefore, the taste difference was ambiguous and the difference was not recognized among the verjuice samples presented to the panelists.

The results of the verjuice color preference questionnaire (Appendix 3) showed that 50% of consumers preferred the color of verjuice from Tfayfihi variety, which is a red variety of grapes, through visual inspection of color, 38% of consumer preferred the color of the black variety verjuice, and 12% of consumers preferred the lighter colored verjuice of Obeideh variety which is a white grape variety. None of the participants in the color preference questionnaire showed a preference for the color of verjuice of Baytamoni variety.

## 4. Conclusion

In this study, four grape varieties used for the production of verjuice were evaluated. The processing method used was based on the most common traditional method collected by a processing survey. Compared to the fresh samples, the verjuice samples in general showed higher polyphenol content and free radical scavenging activity, lower moisture %, and visually clearer product that is less susceptible for precipitation. Thus, through verjuice processing, the waste of grape cluster thinning was converted into a valuable polyfunctional food product that the panelist chose over the popular lemon in some dishes in the preference survey that was conducted.

The four verjuice samples showed a comparable sensory quality; however, Tfayfihi grape variety was preferred in terms of color and it has the highest active polyphenol content. And according to cluster analysis, Baytamoni is the most similar variety to Tfayfihi with respect to physicochemical properties. Therefore, these two varieties could be mixed in verjuice processing and may lead to a similar verjuice product as Tfayfihi verjuice or Baytamoni verjuice product. This is an advantage when either of the two varieties is of limited availability to the producer. More research is required to study the effect of mixing several grape varieties to optimize the sensory and compositional parameters of verjuice. Further evaluation of processing practices should be established so that the quality of verjuice remains stable over shelf-life, especially in terms of sensory attributes.

## Figures and Tables

**Table 1:** Physicochemical properties of processed and fresh unripe grape juice.

Physicochemical property	Grape varieties				
	Verjuice	Tfayfihi	Baytamoni	Black	Obeideh
Density	Fresh (g/cm^3^)	1.00 ± 0.004	1.00 ± 0.004	1.02^∗^ ± 0.002	1.01 ± 0.014
	Processed (g/cm^3^)	1.01^b,c^^∗∗^ ±0.001	1.01^b,c^^∗∗^ ±0.001	1.00^b^ ±0.0004	0.97^a^ ±0.003
Titratable acidity	Fresh (%)	3.79^∗^ ±0.024	3.45^∗^ ±0.023	3.66^∗^ ±0.245	4.09^∗^ ±0.023
	Processed (%)	4.10^b^^∗∗^ ±0.009	3.95^a^ ±0.026	4.51^c^ ±0.031	4.45^c^ ±0.038
pH	Fresh	2.59^∗^ ±0.010	2.53 ± 0.020	2.56^∗^ ±0.006	2.52^∗^ ±0.012
	Processed	2.52^a^^∗∗^ ±0.006	2.55^b^ ±0.010	2.54^b^ ±0.006	2.55^b^ ±0.006
Total soluble solids	Fresh (°Brix)	4.09 ± 0.289	4.38 ± 0.200	4.91^∗^ ± 0.231	3.72^∗^ ± 0.173
	Processed (°Brix)	4.68^ab^^∗∗^ ±0.529	4.68^ab^ ±0.173	5.38^b^ ±0.289	4.45^a^ ±0.289
Color intensity	Fresh	0.223^∗^ ± 0.001	0.468^∗^ ± 0.008	0.400^∗^ ± 0.004	0.231^∗^ ± 0.003
	Processed	0.970^c^^∗∗^ ±0.014	1.180^d^ ±0.007	0.876^b^ ±0.007	0.719 ^a^ ±0.002
Browning index	Fresh	0.243^c^^∗∗^ ±0.005	0.322^d^ ±0.004	0.192^a^ ±0.004	0.202^b^ ±0.003
	Processed	0.428^b^ ±0.061	0.432^b^ ±0.002	0.390^b^ ±0.001	0.228^a^ ±0.003
Total polyphenols	Fresh (mg/L)	341.49^∗^ ± 31.827	445.94^∗^ ± 32.100	492.92^∗^ ± 13.547	274 ± 24.080
	Processed (mg/L)	531.96^b^^∗∗^ ±32.508	667.20^c^ ±38.500	676.10 ^c^ ±6.803	263.37^a^ ±35.707
% of H_2_O_2_ scavenged by polyphenols	Fresh (%)	67.93^∗^ ± 0.951	68.79^∗^ ± 4.515	77.45 ± 1.683	69.12 ± 1.580
	Processed (%)	91.76^b^^∗∗^ ±0.425	91.38^b^ ±2.154	72.88^a^ ±2.948	66.96^a^ ±4.760
Total suspended solids	Fresh (g/L)	271.78^∗^ ± 8.720	217.34^∗^ ± 3.912	250.30^∗^ ± 7.026	201.47^∗^ ± 3.54
	Processed (g/L)	40.05^b^^∗∗^ ±1.329	34.39^a^ ±2.064	40.01^b^ ±2.853	32.27^a^ ±0.454
Moisture percentage	Fresh (%)	96.61^∗^ ± 0.417	96.59^∗^ ± 0.331	95.80^∗^ ± 0.335	95.58^∗^ ± 0.255
	Processed (%)	95.85^b^^∗∗^ ±0.185	95.80^b^ ±0.147	94.58^a^ ±0.420	94.88^a^ ±0.270

^∗^A significant difference exists between the parameter of fresh and the processed sample of the same variety using an independent *t*-test. ^∗∗^Means (of the same parameter between different processed varieties) with different letters are significantly different (*p* = 5%) according to Tukey's HSD test. “a,” “b,” “c,” and “d” are denoted on the means of physiochemical test results of processed verjuice samples, where “a” is denoted to the highest mean value and “d” to the lowest mean value.

## Data Availability

The authors confirm that the data supporting the findings of this study are available within the article and any additional material will be available upon request.

## Supplementary Materials

Supplementary Materials Appendix 1: verjuice processing questionnaire. Appendix 2: verjuice consumption questionnaire. Appendix 3: color preference questionnaire.
