# Supplementary figures and images for: Effect of Four Grape Varieties on the Physicochemical and Sensory Properties of Unripe Grape Verjuice
Source: Int J Food Sci. 2020 Jul 12;2020:6457982. doi: 10.1155/2020/6457982 (PMC7374221; doi:10.1155/2020/6457982)

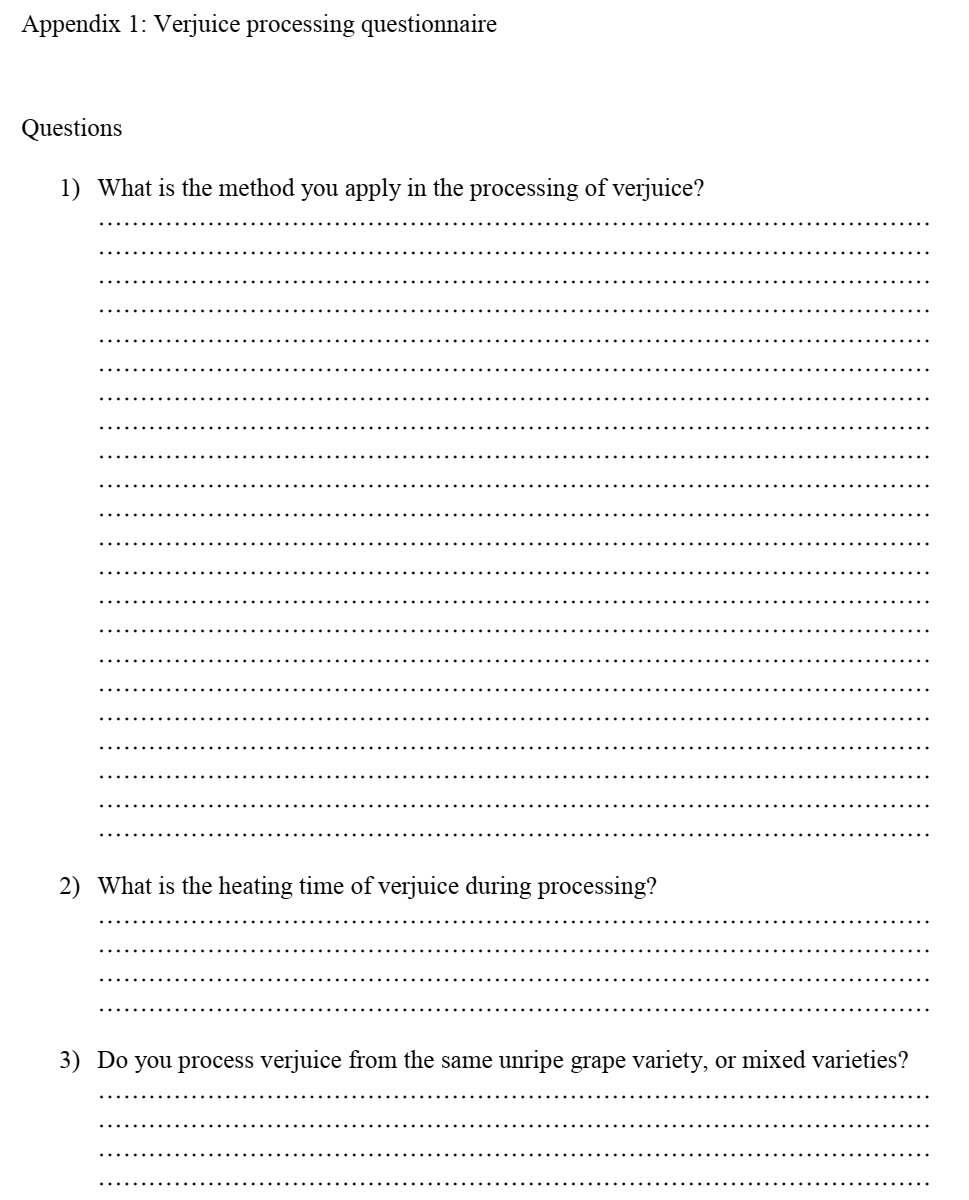


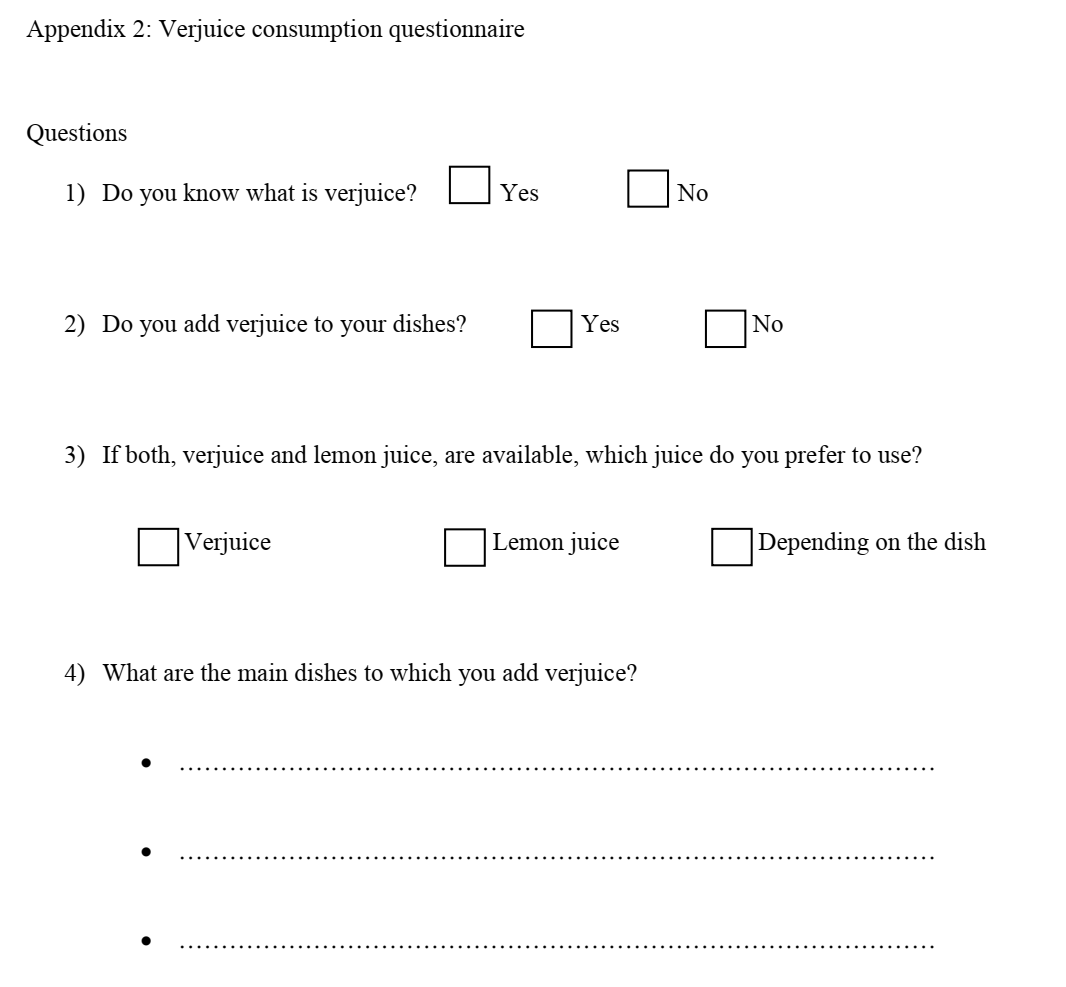


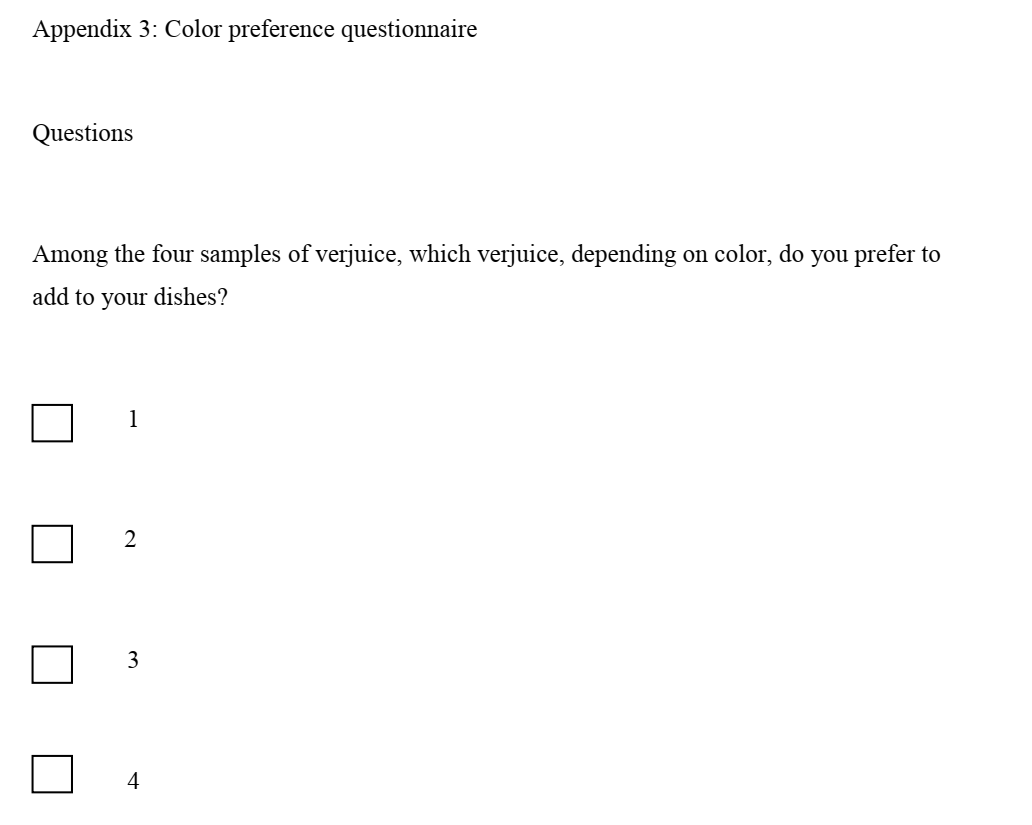

Supplement: Supplementary Materials — Appendix 1: verjuice processing questionnaire. Appendix 2: verjuice consumption questionnaire. Appendix 3: color preference questionnaire. [file 6457982.f1.docx]
